# Fibroblast activation protein targeted therapy using [^177^Lu]FAPI-46 compared with [^225^Ac]FAPI-46 in a pancreatic cancer model

**DOI:** 10.1007/s00259-021-05554-2

**Published:** 2021-09-18

**Authors:** Yuwei Liu, Tadashi Watabe, Kazuko Kaneda-Nakashima, Yoshifumi Shirakami, Sadahiro Naka, Kazuhiro Ooe, Atsushi Toyoshima, Kojiro Nagata, Uwe Haberkorn, Clemens Kratochwil, Atsushi Shinohara, Jun Hatazawa, Frederik Giesel

**Affiliations:** 1 Department of Nuclear Medicine and Tracer Kinetics, Graduate School of Medicine, Osaka University, 2-2 Yamadaoka, Suita, Osaka 565-0871 Japan; 2 Institute for Radiation Sciences, Osaka University, 2-2 Yamadaoka, Suita, Osaka 565-0871 Japan; 3 Core for Medicine and Science Collaborative Research and Education, Project Research Center for Fundamental Sciences, Graduate School of Science, Osaka University, Suita, Osaka Japan; 4 Department of Radiology, Osaka University Hospital, Suita, Osaka Japan; 5 Radioisotope Research Center, Institute for Radiation Sciences, Osaka University, Suita, Osaka Japan; 6 Department of Nuclear Medicine, University Hospital Heidelberg, Heidelberg, Germany; 7 Clinical Cooperation Unit Nuclear Medicine DKFZ, Heidelberg, Germany; 8 Translational Lung Research Center Heidelberg (TLRC), German Center for Lung Research (DZL), Heidelberg, Germany; 9 Department of Chemistry, Graduate School of Science, Osaka University, Toyonaka, Osaka Japan; 10 Research Center for Nuclear Physics, Osaka University, Suita, Osaka Japan; 11 Department of Nuclear Medicine, University Hospital Düsseldorf, Düsseldorf, Germany

**Keywords:** Fibroblast activation protein, Pancreatic cancer, FAPI, Lutetium, Actinium

## Abstract

**Purpose:**

Fibroblast activation protein (FAP), which has high expression in cancer-associated fibroblasts of epithelial cancers, can be used as a theranostic target. Our previous study used ^64^Cu and ^225^Ac-labelled FAP inhibitors (FAPI-04) for a FAP-expressing pancreatic cancer xenograft imaging and therapy. However, the optimal therapeutic radionuclide for FAPI needs to be investigated further. In this study, we evaluated the therapeutic effects of beta-emitter (^177^Lu)-labelled FAPI-46 and alpha-emitter (^225^Ac)-labelled FAPI-46 in pancreatic cancer models.

**Methods:**

PET scans (1 h post injection) were acquired in PANC-1 xenograft mice (*n* = 9) after the administration of [^18^F]FAPI-74 (12.4 ± 1.7 MBq) for the companion imaging. The biodistribution of [^177^Lu]FAPI-46 and [^225^Ac]FAPI-46 were evaluated in the xenograft model (total *n* = 12). For the determination of treatment effects, [^177^Lu]FAPI-46 and [^225^Ac]FAPI-46 were injected into PANC-1 xenograft mice at different doses: 3 MBq (*n* = 6), 10 MBq (*n* = 6), 30 MBq (*n* = 6), control (*n* = 4) for [^177^Lu]FAPI-46, and 3 kBq (*n* = 3), 10 kBq (*n* = 2), 30 kBq (*n* = 6), control (*n* = 7) for [^225^Ac]FAPI-46. Tumour sizes and body weights were followed.

**Results:**

[^18^F]FAPI-74 showed rapid clearance by the kidneys and high accumulation in the tumour and intestine 1 h after administration. [^177^Lu]FAPI-46 and [^225^Ac]FAPI-46 also showed rapid clearance by the kidneys and relatively high accumulation in the tumour at 3 h. Both [^177^Lu]FAPI-46 and [^225^Ac]FAPI-46 showed tumour-suppressive effects, with a mild decrease in body weight. The treatment effects of [^177^Lu]FAPI-46 were relatively slow but lasted longer than those of [^225^Ac]FAPI-46.

**Conclusion:**

This study suggested the possible application of FAPI radioligand therapy in FAP-expressing pancreatic cancer. Further evaluation is necessary to find the best radionuclide with shorter half-life, as well as the combination with therapies targeting tumour cells directly.

**Supplementary Information:**

The online version contains supplementary material available at 10.1007/s00259-021-05554-2.

## Introduction

The stroma, which comprises up to 90% of tumour mass, promotes tumour growth, migration, and progression. The fibroblast activation protein (FAP) is highly expressed in cancer-associated fibroblasts (CAFs) of the stroma of many epithelial cancers and is associated with poor prognosis [1–3]. In contrast, low FAP expression is found in normal tissues. Therefore, FAP is an excellent target for the imaging and therapy. FAP inhibitors (FAPI) are used for theranostics in oncology [4–6]. In previous studies, [^68^ Ga]-labelled FAPI positron emission tomography (PET)/computed tomography (CT) were proven to be effective in the clinical diagnostics of various cancers [7–9]. [^99m^Tc]-labelled FAPI derivatives were also synthesized successfully for single photon emission computed tomography imaging [6]. However, reports regarding the therapeutic applications of FAPI are relatively limited. Lindner et al. used [^90^Y]FAPI-04 for the targeted therapy in a breast cancer patient resulting in pain reduction [10]. Our previous study labelled FAPI-04 with ^225^Ac, an alpha particle emitter with a half-life of 10 days for its first decay, and investigated the therapeutic effects of [^225^Ac]FAPI-04 in FAP-expressing human pancreatic cancer [11]. However, FAPI showed rapid excretion via the kidneys, and its biological half-life did not match the physical half-life of ^225^Ac. Therefore, it is necessary to compare the therapeutic effects of FAPI with improved tumour retention, to investigate a better combination of its kinetics and physical decay. In this study, we used ^177^Lu, a beta emitter with a half-life of 6.7 days, and ^225^Ac to label FAPI-46 for the targeted therapy and [^18^F]FAPI-74 PET companion imaging. The purpose of this study was to compare the therapeutic effects of [^177^Lu]-labelled and [^225^Ac]-labelled FAPI in FAP-expressing pancreatic cancer xenografts.

## Materials and methods

### *Preparation of [*^*18*^*F]FAPI-74, [*^*177*^*Lu], and [*^*225*^*Ac]FAPI-46 solutions*

The precursor molecules of FAPI-46 and FAPI-74 were obtained from the Heidelberg University based on a material transfer agreement for collaborative research. [^18^F]FAPI-74 was produced following the methods of previous reports [12]. [^18^F]fluoride eluted with 0.3 mL of 0.5 M sodium acetate (pH 3.9) from an anion-exchange cartridge (Sep-Pak Accell Plus QMA Plus Light Cartridge, Waters, Milford, MA) was mixed with 0.3 mL of dimethyl sulfoxide (FUJIFILM Wako Pure Chemical, Osaka, Japan) and 6 µL of 10 mM aluminum chloride at room temperature for 5 min. Twenty microliters of 4 mM FAPI-74 and 4 µL of 20% ascorbic acid were then added, and fluorination was performed at 95 °C for 15 min. The mixture was diluted with 10 mL of 0.9% saline, and [^18^F]FAPI-74 was captured by passing this diluted solution through a hydrophilic-lipophilic balance cartridge (Oasis HLB Plus Light Cartridge, Waters, Milford, MA). After washing the cartridge with 3 mL of 0.9% saline, [^18^F]FAPI-74 was recovered with 0.3 mL of ethanol into a vial containing 2.7 mL of 0.9% saline. The chemical structure of [^18^F]FAPI-74 is shown in Sup Fig. 1a.

Lutetium-177 chloride ([^177^Lu]LuCl_3_, 1,110 MBq/mL) dissolved in 0.05 M hydrochloric acid was purchased from Polatom (Otwock, Poland). Actinium-225 nitrate ([^225^Ac]Ac(NO_3_)_3_, solid) was sourced from the Institute of Material Research at Tohoku University and the Japan Atomic Energy Agency, and was dissolved in 0.2 M ammonium acetate.

[^177^Lu]FAPI-46 was prepared from the mixture of solutions of 175 µL of 1 mM FAPI-46, 408 µL of 0.2 M sodium acetate, 100 µL of 10% ascorbic acid, and 555 µL of [^177^Lu]LuCl_3,_ reacted at 50℃, for 60 min. The solution was diluted with 0.9% saline, and was used for the animal studies without further purification. The chemical structure of [^177^Lu]FAPI-46 is shown in Sup Fig. 1b.

[^225^Ac]labelled FAPI-46 was prepared as per the method provided in a previous paper [11]. A mixture of 30 µL of 1 mM FAPI-46, 100 µL of 0.2 M ammonium acetate, 100 µL of 7% sodium ascorbate, and 200 µL of ^225^Ac solution (300 kBq) was obtained at 80℃ after 2 h. The solution was diluted with 0.9% saline, and was used for the animal studies without further purification. The chemical structure of [^225^Ac]FAPI-46 is shown in Sup Fig. 1c.

### *Preparation of the animals*

The human pancreatic cell line, PANC-1, was obtained from the American Type Culture Collection (Manassas, VA, USA). The cells were cultured in RPMI1640 medium with l-glutamine and Phenol Red (FUJIFILM Wako Pure Chemical, Osaka, Japan), supplemented with 10% heat-inactivated fetal bovine serum and 1% penicillin–streptomycin.

Male nude mice (BALB/cSlc-nu/nu) were purchased from Japan SLC Inc. (Hamamatsu, Japan). Animals were housed under a 12-h light/12-h dark cycle and allowed free access to food and water. The mice were injected with PANC-1 cells (1 × 10^7^ cells) in a mixture of phosphate-buffered saline and Matrigel (0.1 mL, 1:1; BD Biosciences, Franklin Lakes, NJ, USA). Tumour xenograft models were evaluated 3 weeks after transplantation of PANC-1 cells (tumour volume = 716 ± 304 mm^3^). Euthanasia was performed in the following conditions: (1) when the animals had unbearable suffering, (2) when a significant decrease in activity or a marked decrease in food and water intake was observed, and (3) at the end of the observation period (up to 44 days for [^177^Lu]FAPI-46 and 32 days for [^225^Ac]FAPI-46). Euthanasia was performed by deep anesthesia using isoflurane inhalation.

### *[*^*18*^*F]FAPI-74 PET imaging and analysis*

PET images were acquired with a small animal PET scanner (Siemens Inveon PET/CT) 3 weeks after the implantation in PANC-1 xenograft mice (9 weeks old, body weight = 25.3 ± 1.2 g, *n* = 9). Under 2% isoflurane anesthesia, [^18^F]FAPI-74 (12.4 ± 1.7 MBq) was injected in the tail vein. Dynamic PET scans (scan duration = 70 min, *n* = 2) were started simultaneously with the bolus injection. Static PET scans (scan duration = 10 min, *n* = 7) were performed 1 h after injection, followed by a CT scan. PET data were reconstructed into 2-min frames in the dynamic PET scan (2 min × 35 frames) and one frame in the static PET scan by three-dimensional ordered-subset expectation–maximization (16 subsets, 2 iterations), with attenuation and scatter correction. Regions of interest were drawn on the muscle, heart, lungs, liver, gallbladder, kidneys, intestine, and tumour. The mean standardized uptake values (SUVmean) and maximum standardized uptake values (SUVmax) were measured using PMOD (Version 4.0).

### *Biodistribution of [*^*177*^*Lu]FAPI-46 and [*^*225*^*Ac]FAPI-46 in mice*

[^177^Lu]FAPI-46 (3.3 ± 0.1 MBq) and [^225^Ac]FAPI-46 (12.5 ± 0.7 kBq) were injected into PANC-1 xenograft mice (9 weeks old, body weight = 23.9 ± 0.9 g, *n* = 12). After euthanasia by deep inhalation anesthesia with isoflurane, the brain, thyroid gland, salivary glands, lungs, heart, liver, spleen, pancreas, stomach, small intestine, large intestine, kidneys, bone (femur), bladder, testis, tumour, blood, and urine were removed and weighed at 3 h and 24 h. Radioactivity was also measured using a gamma counter (2480 Wizard^2^ Gamma Counter, Perkin Elmer, USA).

### *Treatment effect of [*^*177*^*Lu]FAPI-46 and [*^*225*^*Ac]FAPI-46 in the mice*

[^177^Lu]FAPI-46 and [^225^Ac]FAPI-46 were injected into PANC-1 xenograft mice via the tail vein (9 weeks old, body weight = 22.7 ± 2.1 g). Mice injected with [^177^Lu]FAPI-46 were divided into four groups according to the injected dose: 3 MBq (3.2 ± 0.1 MBq, *n* = 6), 10 MBq (10.2 ± 0.5 MBq, *n* = 6), 30 MBq (30.5 ± 2.7 MBq, *n* = 6) and control (*n* = 4) groups. Mice injected with [^225^Ac]FAPI-46 were divided into four groups: 3 kBq (2.9 ± 0.0 kBq, *n* = 3), 10 kBq (8.5 ± 1.1 kBq, *n* = 2), 30 kBq (30.5 ± 0.7 kBq, *n* = 6) and control (*n* = 7) groups. The tumour size and body weight were measured with a caliper using the elliptical sphere model calculation, three times per week during the observation period.

### *Immunohistochemistry and histological analysis*

All mice were killed after [^18^F]FAPI-74 PET imaging, and tumour xenografts were removed. Immunohistochemical staining was performed using anti-FAP alpha antibody (ab53066; Abcam, Cambridge, UK), and the Dako EnVision + System—HRP Labelled Polymer Anti-Rabbit (K4003) (DAKO Corp., Glostrup, Denmark). To evaluate toxicity, the kidneys were removed after the mice treated with [^177^Lu]FAPI-46 and [^225^Ac]FAPI-46 were sacrificed. The tissues were fixed in 10% neutral buffered formalin solution for paraffin blocks and stained with hematoxylin and eosin (H&E). Tumour blocks in all mice were also stained with H&E.

### *Statistical analysis*

Data were expressed as the mean ± standard deviation. Comparisons among the four groups were performed using an unpaired *t* test in Microsoft Excel (version 2016) with Bonferroni correction, and *p* < 0.05 were considered statistically significant.

## Results

**Fig. 1:**
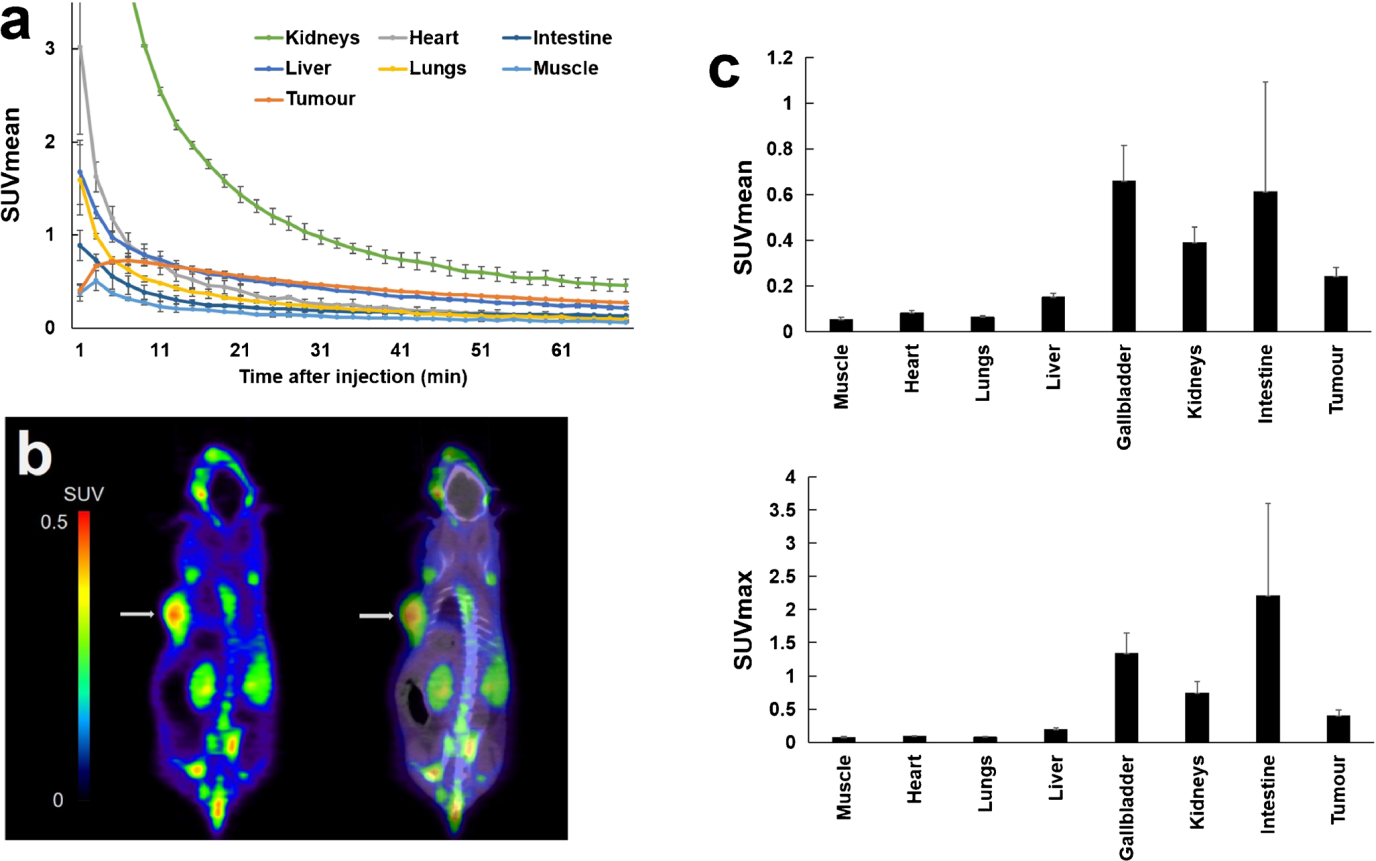
**a** Time-activity curves of [^18^F]FAPI-74 in PANC-1 tumour and normal organs. **b** Static coronal PET imaging (left) and PET/CT fusion imaging (right) of [^18^F]FAPI-74 (1 h post-administration) in PANC-1 xenograft mice. Arrows revealed tumour xenograft on the left side. **c** The SUVmean (upper) and SUVmax (lower) in the tumour and normal organs. The high uptake by the gallbladder and kidneys was due to the excretion through bile and urine

**Fig. 2:**
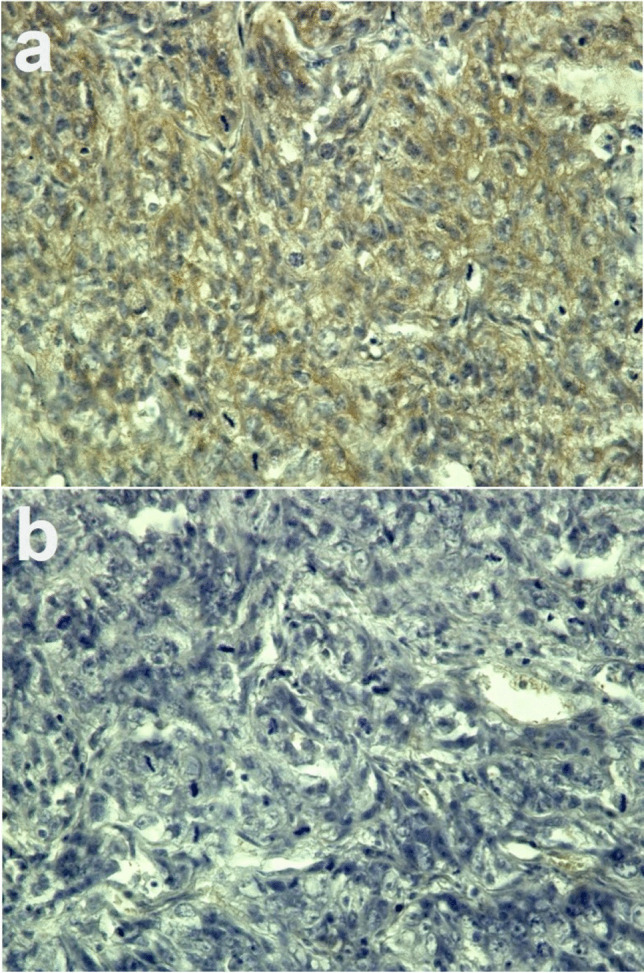
**a** Immunohistochemical staining of fibroblast activation protein (FAP) in PANC-1 xenograft and **b** negative control without primary antibody (magnification × 400). FAP expression was observed in the tumour stroma

The time-activity curve of the PANC-1 tumour and the normal organs on [^18^F]FAPI-74 PET are shown in Fig. 1a. [^18^F]FAPI-74 was cleared rapidly by the kidneys but washout from the tumour occurred slowly. A static PET image is shown in Fig. 1b. The SUVmean of static scans were 0.24 ± 0.04 in the tumour, 0.05 ± 0.01 in the muscle, 0.08 ± 0.01 in the heart, 0.14 ± 0.02 in the liver, 0.66 ± 0.15 in the gallbladder, 0.61 ± 0.48 in the intestine, and 0.39 ± 0.07 in the kidneys (Fig. 1c). The accumulation in the tumour was significantly higher than in most organs at 1 h post-injection. Immunohistochemical staining showed FAP expression in the stroma of PANC-1 xenografts (Fig. 2).

**Fig. 3:**
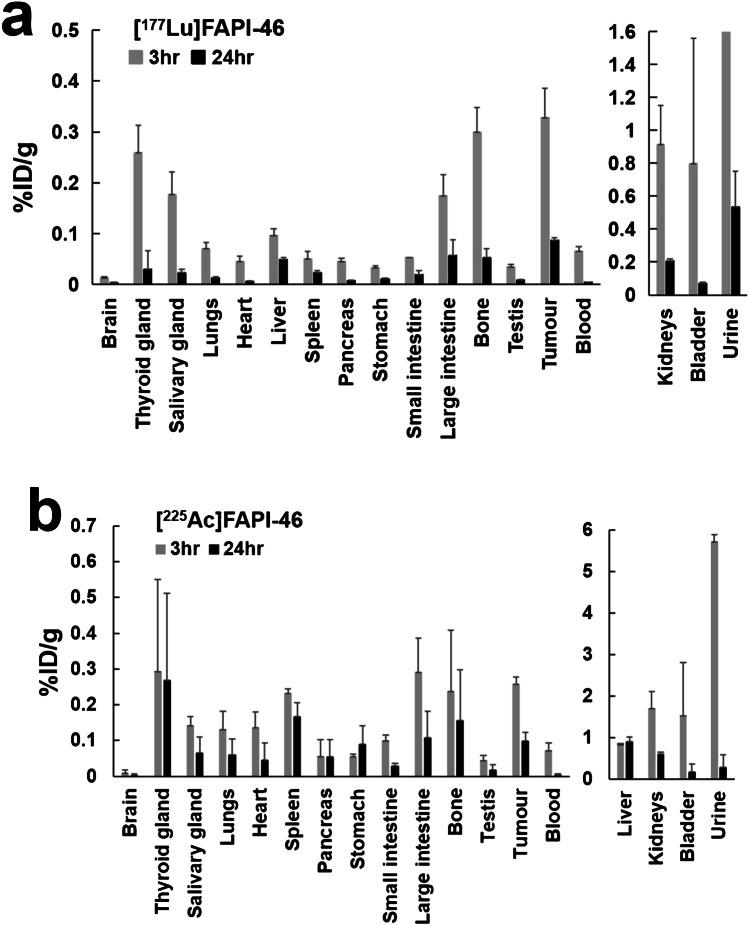
The %ID/g of **a** [^177^Lu]FAPI-46 and **b** [^225^Ac]FAPI-46 in the PANC-1 xenograft mice at 3 h and 24 h post-administration. (%ID/g of the urine of [^177^Lu]FAPI-46 was 21.2 ± 15.3% at 3 h post-administration.)

The biodistribution of [^177^Lu]FAPI-46 and [^225^Ac]FAPI-46 is shown in Fig. 3. Most organs showed fast clearance between 3 and 24 h post-administration. While a relatively high tracer accumulation was seen in the tumour, large intestine, and bone at 24 h post-injection of [^177^Lu]FAPI-46, [^225^Ac]FAPI-46 showed a relatively higher residence time in the tumour, spleen, liver, stomach, small intestine, and large intestine.

**Fig. 4:**
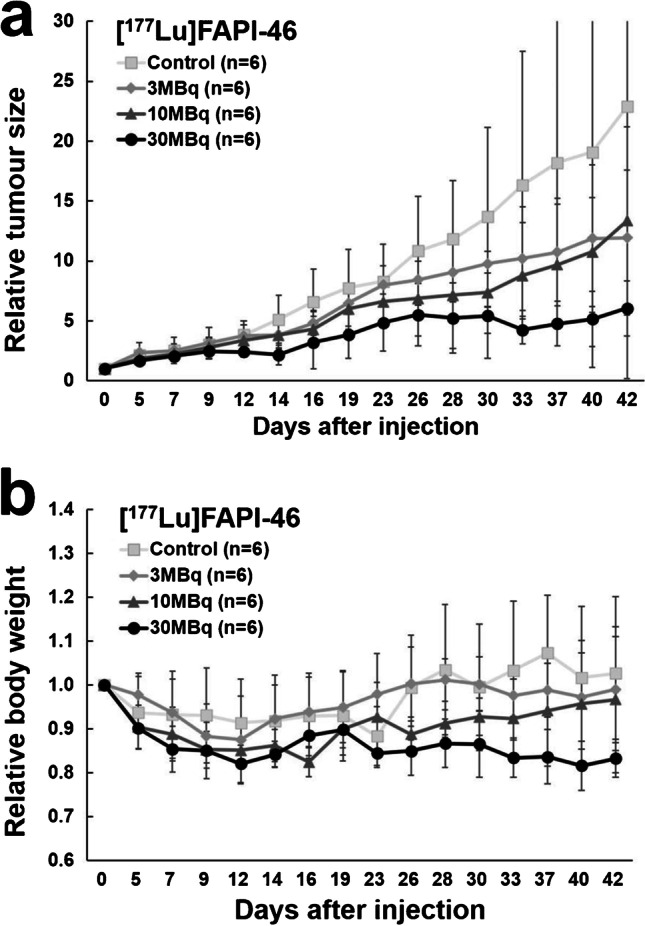
Changes in **a** the relative tumour size and **b** the relative body weight in PANC-1 xenograft mice treated with [^177^Lu]FAPI-46

The changes in the tumour size and body weight after administration with [^177^Lu]FAPI-46 are shown in Fig. 4 and the relative tumour size of each animal is shown in Sup Fig. 2a. Tumour growth showed an inhibitory trend after administration, although the changes were not statistically significant, by the multiple comparisons across the doses. The tumour-suppressive effects in the 30 MBq group were observed 9 days after administration of [^177^Lu]FAPI-46, while the therapeutic effects in 3 MBq and 10 MBq groups were slower and were seen until day 12. The relative ratio of the tumour size in the 3 MBq, 10 MBq, and 30 MBq groups was 0.62, 0.56, and 0.27 at day 40, respectively, compared to the control group. The body weight in the 10 MBq and 30 MBq groups showed a slight decrease without statistical significance, compared to the controls (Fig. 4b).

**Fig. 5:**
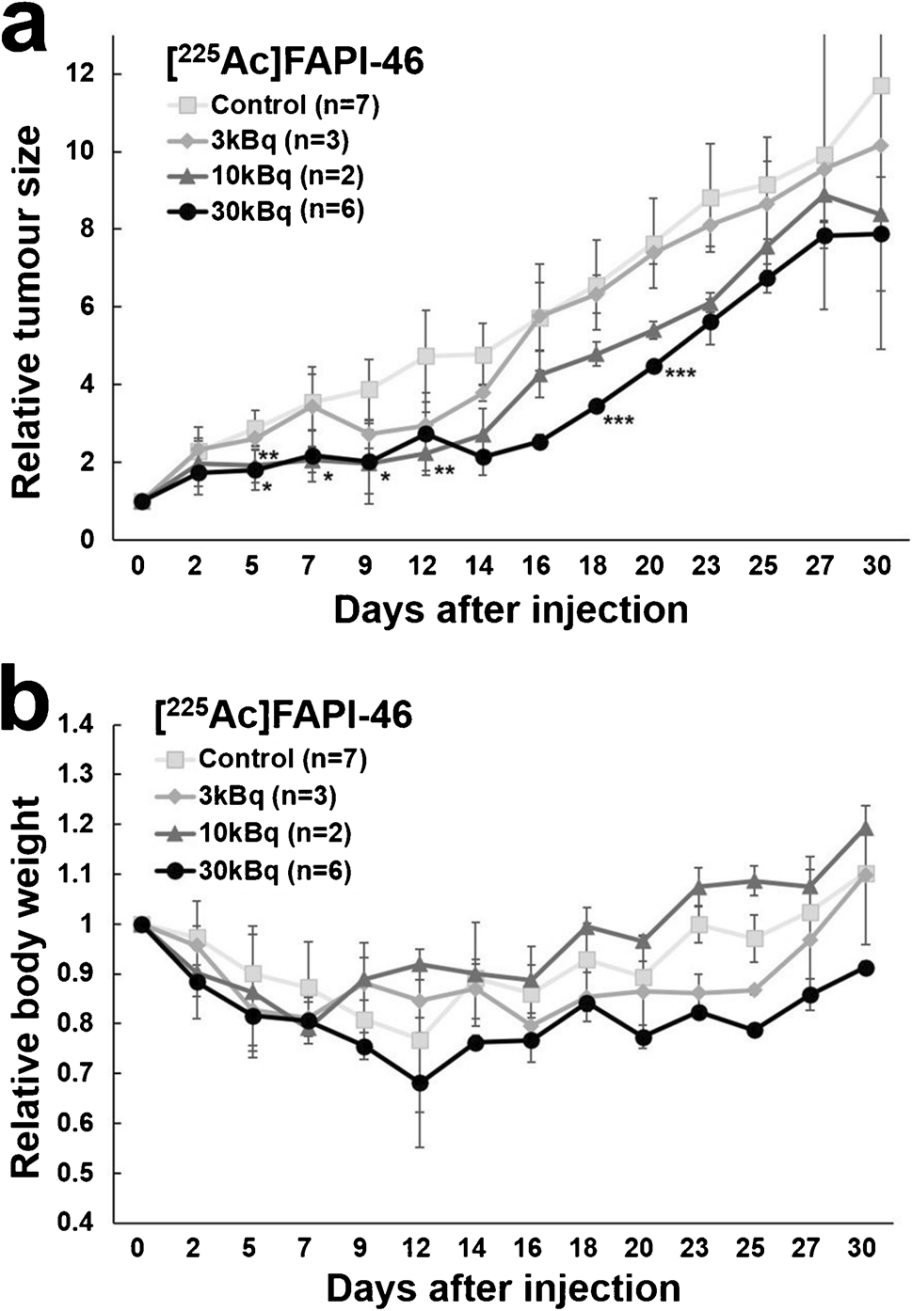
Changes of **a** the relative tumour size and **b** the relative body weight in PANC-1 xenograft mice treated with [^225^Ac]FAPI-46. (*p < 0.05 between 10 kBq and control group; **p < 0.05 between 30 kBq and control group; ***p < 0.05 between 3 and 30 kBq group.)

The results after the administration of [^225^Ac]FAPI-46 are shown in Fig. 5 and Sup Fig. 2b. The tumour growth was suppressed immediately after treatment in the 10 kBq and 30 kBq groups, while the tumour-suppressive effects in the 3 kBq group were very mild. The tumour size of the 30 kBq groups was significantly smaller than those in the control group on days 5–9 and day 25. The body weight in all the groups showed a decreasing trend in the first week while the 3 kBq and 10 kBq groups showed recovery after day 7.

**Fig. 6:**
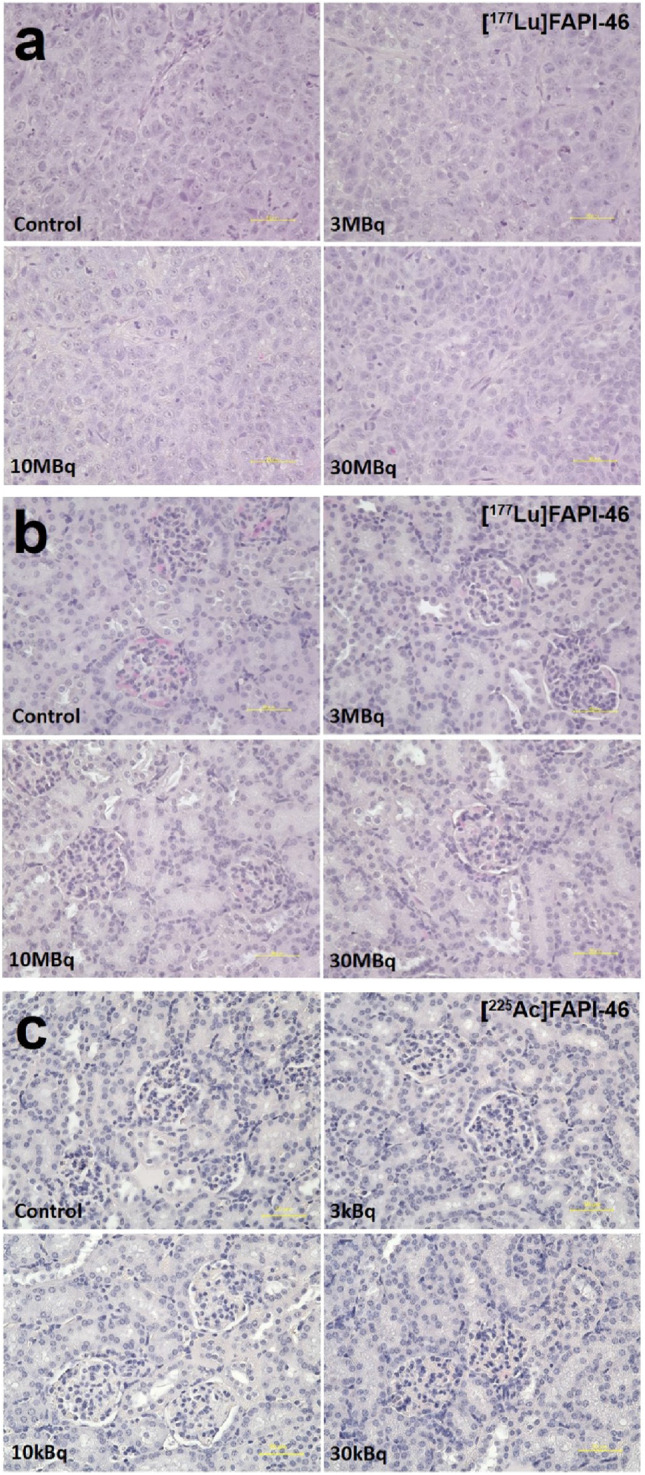
Histological changes evaluated by hematoxylin and eosin staining in **a** the tumour and **b** the kidney at day 44 after the administration of [^177^Lu]FAPI-46, and **c** the kidney at day 32 after the administration of [^225^Ac]FAPI-46. No significant difference was observed both in the tumour and the kidney compared to controls. Yellow bar indicates 50 μm

H&E staining of the tumours and kidneys are shown in Fig. 6. No histological changes were observed in the kidneys of mice injected with [^177^Lu]FAPI-46 on day 44 and mice injected with [^225^Ac]FAPI-46 on day 32.

## Discussion

The present study showed a rapid clearance of [^177^Lu]FAPI-46 and [^225^Ac]FAPI-46 from most normal organs and tumours, but relatively high accumulation was observed at 3 h post-injection in the PANC-1 tumour model. Tumour-suppressive effects were observed in both PANC-1 xenograft mice treated with [^177^Lu]FAPI-46 and [^225^Ac]FAPI-46, respectively. [^177^Lu]FAPI-46 showed mild but more prolonged therapeutic effects as compared to [^225^Ac]FAPI-46. We also performed [^18^F]FAPI-74 PET in PANC-1 xenograft mice and confirmed the high uptake in the tumour as well as the confirmation of FAP expression in the tumour stroma by immunohistochemistry.

We demonstrated the effectiveness of alpha therapy for FAP-expressing pancreatic cancer using [^225^Ac]FAPI-04 in a previous study [11]. [^225^Ac]FAPI-04 was thought to irradiate tumour cells by the alpha particles emitted from CAFs in the stroma. However, the alpha irradiation also has affects on CAFs, the primary site of accumulation, which are supporting tumour progression. Since beta particles have a more extended range in tissue compared to alpha particles, beta irradiation may reach tumour cells more homogeneously compared to alpha irradiation. Thus, we used FAPI-46 labelled with ^177^Lu, a beta emitter, for PANC-1 xenograft mice in the present study. Previous studies reported a rapid internalization of [^177^Lu]-labelled FAPI derivatives into HT-1080-FAP cells [4] and a high uptake in HT-1080-FAP tumour-bearing mice [10, 13]. In the present study, we also found a relatively high accumulation of [^177^Lu]FAPI-46 in PANC-1 xenografts, which is considered to target FAP mainly expressed in the stroma.

In the present study, we found that [^177^Lu]FAPI-46 suppresses tumour growth. Meanwhile, other beta-emitters, such as ^90^Y, ^188^Re, and ^153^Sm, -labelled with FAPI derivatives were administered in humans without serious side effects [6, 10, 14], suggesting the potential clinical application of [^177^Lu]FAPI-46. Compared with [^177^Lu]FAPI-46, [^225^Ac]FAPI-46 showed faster therapeutic effects in PANC-1 xenograft mice with a shorter duration. The tumour size in mice treated with a high dose of [^177^Lu]FAPI-46 started to reduce at 9 days after administration, with a slower growth rate than the control group. In contrast, the tumour growth reduced immediately after administering a high dose of [^225^Ac]FAPI-46, while regrowth began at day 12 with the same tumour growth speed as in the control group. However, in a previous study, ^225^Ac showed a lower survival rate of cells compared to cells treated with ^177^Lu [15], according to more fatal double-strand breaks (DSBs) induced by alpha particles [16, 17]. Meanwhile, [^225^Ac]PSMA-617 was effective in metastatic prostate cancer patients refractory to [^177^Lu]PSMA-617 [18, 19]. We speculated that the reason for the difference seen in our study was due to the fact that the target cells of [^177^Lu]FAPI-46 and [^225^Ac]FAPI-46 were CAFs in the stroma as opposed to tumour cells. Stroma cells can tolerate a more fatal environment than other cells and are more radioresistant [20, 21]. However, the effects of alpha irradiation on tumour stromal cells remain to be clarified. Due to a heterogeneous distribution of the stroma and tumour cells causing a heterogeneous dose distribution, it might be difficult for alpha particles to reach the tumour cells sufficiently. The tumour cells irradiated by ^225^Ac caused death due to DSBs, while the tumour cells without irradiation survived and recovered. In contrast, the tumour cells are more likely to be irradiated by beta emission from [^177^Lu]FAPI-46 but with lower cell-killing properties. In the present study, the marginal superiority of [^177^Lu]FAPI-46 was observed compared to [^225^Ac]FAPI-46 and possibly due to the wide effective area by cross-fire effect of beta emission from ^177^Lu accompanied with bystander effects as well as the inefficient energy transfer by alpha emission of ^225^Ac from the stroma.

In the present study, the therapeutic effects of [^177^Lu]FAPI-46 and [^225^Ac]FAPI-46 were rather limited, with some of the tumour-suppressive effects being not significant compared to the control group. When we compare these therapeutic effects with previous reports using other compounds with a similar administered dose, such as [^177^Lu]DOTATATE for neuroendocrine tumour xenografts, and [^177^Lu]PSMA-617 for prostate cancer, the anti-tumour effects were inferior in our study [22–24]. A characteristic of FAPI is its quick distribution, but retention in the tumour was inferior to that of other compounds. Lindner et al. developed the FAPI compounds with improved retention, and FAPI-46 shows a better retention compared to FAPI-04 [13]. However, dramatic improvement of retention is not an easy task and the biological half-life of FAPI is short, compared to the long physical half-life of ^177^Lu and ^225^Ac. Thus, the possible strategy is to improve treatment effect is injecting high radioactivity with shorter half-life radionuclide. Radionuclides with a shorter half-life, such as ^188^Re (half-life = 17.0 h) or ^211^At (half-life = 7.2 h) that reaches tumour with high radioactivity at an early time of administrations, maybe optimal for FAPI therapy by increasing the local dose. Another alpha therapy targeting cancer-specific LAT1, which also showed fast clearance through urine, chose short-half-life radionuclide ^211^At for labelling [25]. Although the procedure for labelling FAPI with ^211^At has not been established yet, [^188^Re]-labelled FAPI was synthesized successfully recently and administrated clinically [6]. Therapeutic effects of [^188^Re]- and [^211^At]-labelled FAPI should be compared in a future study to investigate these nuclides as more suitable option for FAPI treatment. In addition, combination with other therapies targeting tumour cells directly can be considered to improve the anti-tumour effect of FAPI radioligand therapy.

The minimum dose of [^177^Lu]FAPI-46 (3 MBq per mouse) was decided according to the recommended dose of [^177^Lu]DOTATATE in clinical practice (7.4 GBq), which is about 2.8 MBq for mice based on body weight conversion. The dose of [^177^Lu]FAPI-46 was increased until 30 MBq which is the lethal dose of [^177^Lu]LuCl_3_ in PANC-1 xenograft mice (data not shown). We injected 34 kBq of [^225^Ac]FAPI-04 per mouse in our previous study. However, this dose is relatively very high compared to ^225^Ac-PSMA-617 therapy (50–200 kBq/kg) in humans [11]. Considering about the potential side effects, we set the maximum administration activity of [^225^Ac]FAPI-46 as 30 kBq per mouse in the present study.

[^18^F]FAPI-74 showed a high uptake in the joints, and similar uptakes in the joints and bone were reported in the use of [^18^F]FGlc-FAPI in FAP-expressing xenograft models [5]. They also reported that possible specific binding in the joints and bones by blocking experiments although they are reported to be low in [^68^ Ga]FAPI-04 PET. [^177^Lu]FAPI-46 and [^225^Ac]FAPI-46 also showed relatively high uptake in the bone. In the present study, we assumed that these uptakes in mice may be due to the binding of radio-labelled FAPIs to the protein in the murine synovial fluid in the joints, since no high uptake was found in human joints [12].

In the present study, [^225^Ac]FAPI-46 showed high accumulation in the liver, whereas the uptake of [^177^Lu]FAPI-46 in the liver was low. A previous study also reported an increased accumulation of [^225^Ac]DOTATOC in the liver compared to [^177^Lu]DOTATOC [26]. The difference was thought to be due to the distribution of free ^225^Ac since a high uptake of released ^225^Ac in the liver was found in mice [27], suggesting better in vivo stability of [^177^Lu]FAPI-46.

A slight decrease of body weight in control group was found, and it may due to the stress caused by the change of housing conditions. Both mice in [^177^Lu]FAPI-46 and [^225^Ac]FAPI-46 groups also showed a decrease of body weight, which is a sign of worry for potential clinical application. Extended single-dose toxicity study in normal mice should be performed in the future study to evaluate the possible side effects [28]. Renal toxicity was observed in patients treated with [^177^Lu]DOTATATE. Renal dysfunction might occur years after [^177^Lu]DOTATATE therapy, even under kidney protection [29, 30]. However, no histological change was observed in the kidneys after administering [^177^Lu]FAPI-46 and [^225^Ac]FAPI-46 in the present study. Although further evaluation should be performed in future studies, our results suggest the clinical feasibility of [^177^Lu]FAPI-46 and [^225^Ac]FAPI-46 treatment.

This study had several limitations. First, we used only one cell line, PANC-1, for the evaluation. However, stroma formation may be different from the tumour stroma in the patients. Therefore, patient-derived xenograft (PDX) models or other non-PDX models with different FAP expression should be used in future work for the better clinical translation. Second, the sample size of [^225^Ac]FAPI-46 was insufficient because of the limited supply of ^225^Ac. Third, we did not determine the maximum tolerated dose (MTD) of [^177^Lu]FAPI-46 and [^225^Ac]FAPI-46 in PANC-1 model, which helps to determine therapeutic window in the future clinical applications. We need to perform the toxicity study using normal mice to evaluate the possible side effects to determine the MTD in mice [28].

## Conclusion

This study revealed therapeutic effects of [^177^Lu]FAPI-46 and [^225^Ac]FAPI-46 in PANC-1 xenografts, while the impact of [^177^Lu]FAPI-46 appeared slow but lasted longer. Beta therapy and alpha therapy targeting FAP can be a potential treatment for pancreatic cancers and needs further evaluation to find the best combination of fast FAP kinetics and physical decay of the radionuclide as well as the combination with therapies targeting tumour cells.

## Supplementary Information

Below is the link to the electronic supplementary material.

Supplementary file1 (DOCX 135 KB)

Supplementary file2 (DOCX 381 KB)

## Data Availability

Data available on request.
