# Supplementary figures and images for: Fibroblast activation protein targeted therapy using [177Lu]FAPI-46 compared with [225Ac]FAPI-46 in a pancreatic cancer model
Source: Eur J Nucl Med Mol Imaging. 2021 Sep 18;49(3):871–80. doi: 10.1007/s00259-021-05554-2 (PMC8803706; doi:10.1007/s00259-021-05554-2)

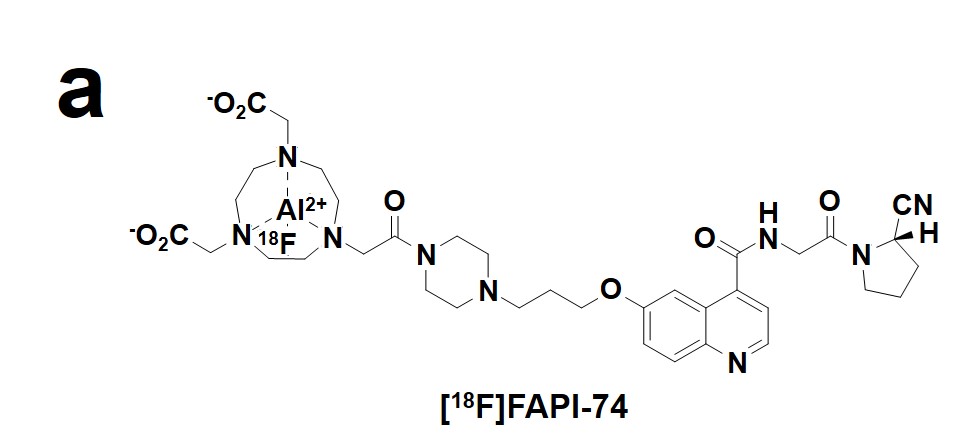

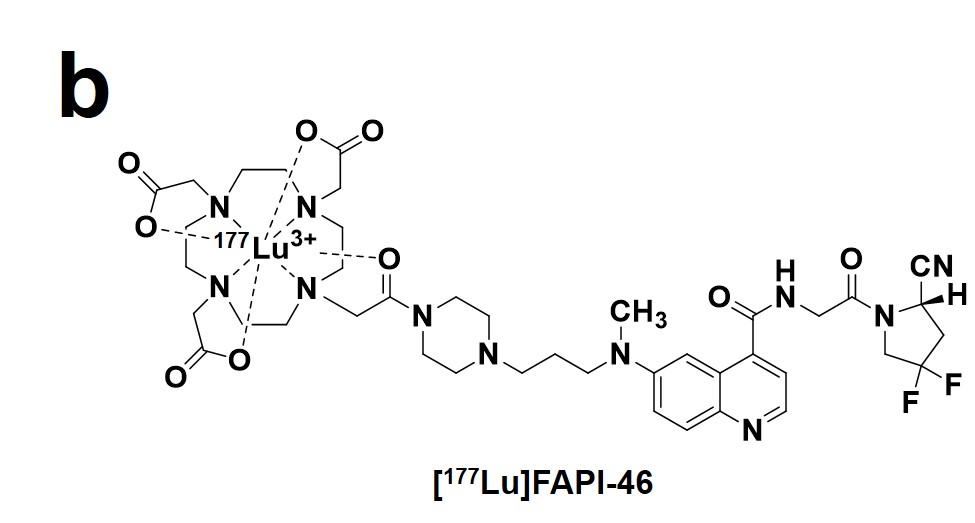

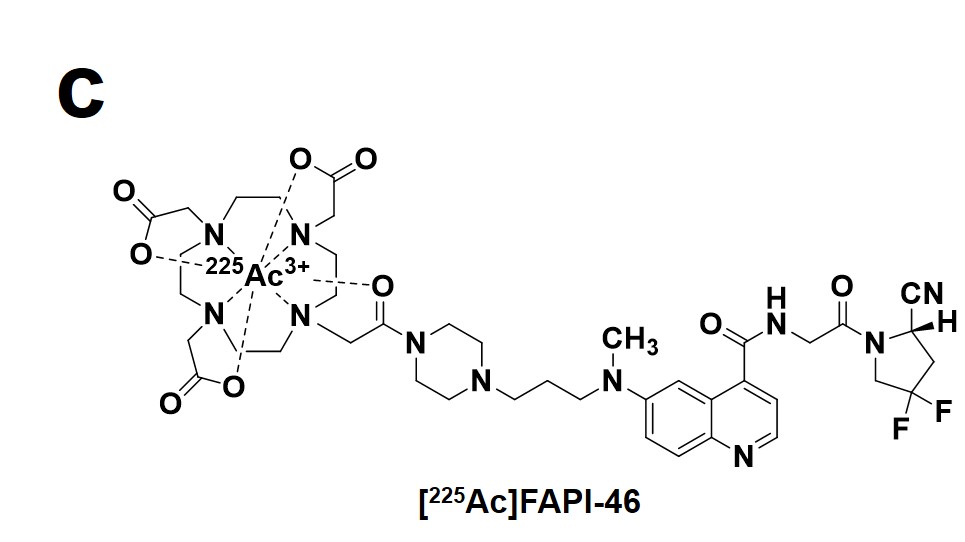

Supplement: Supplementary file 1 — Supplementary file1 (DOCX 135 KB) [file 259_2021_5554_MOESM1_ESM.docx]

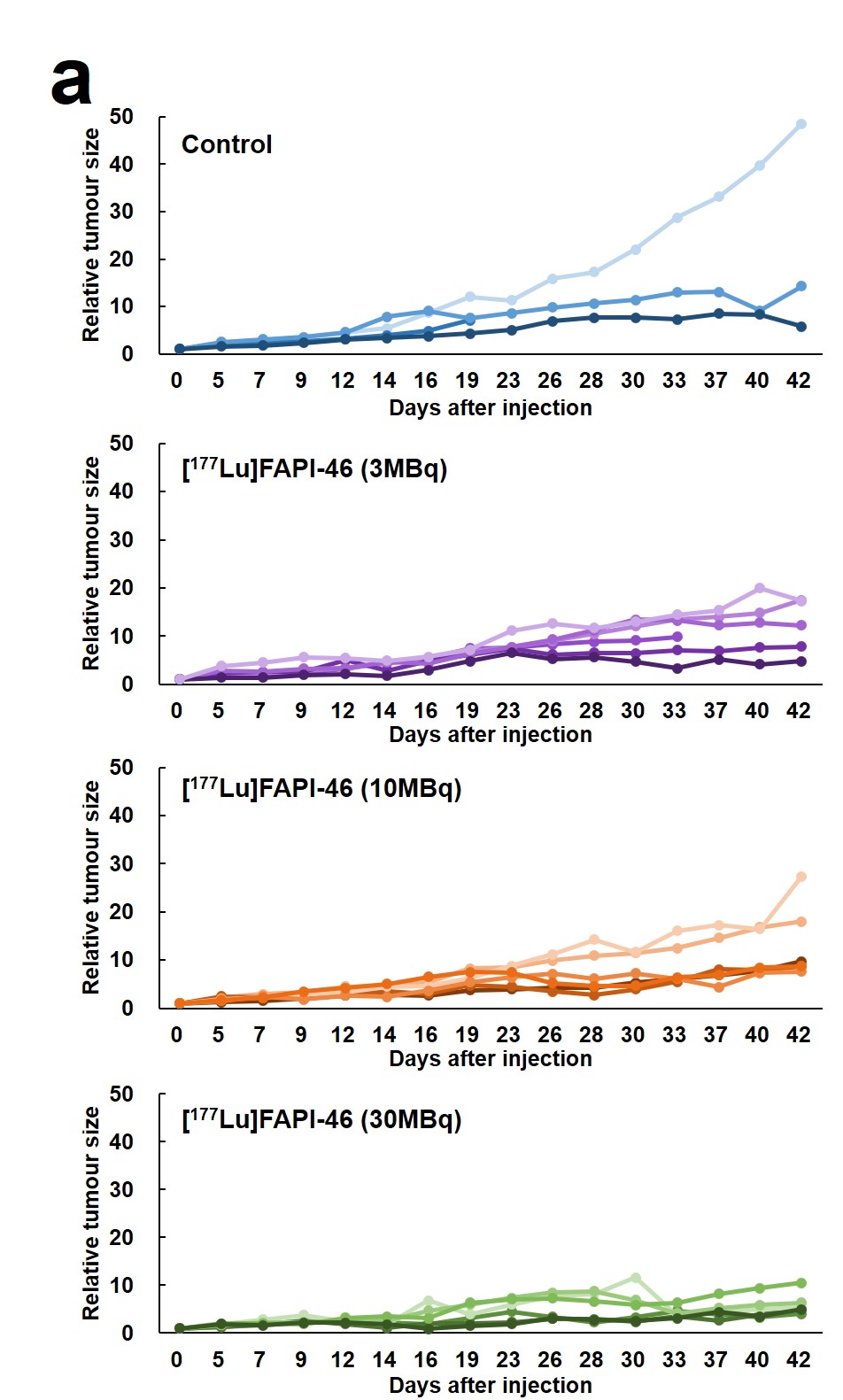

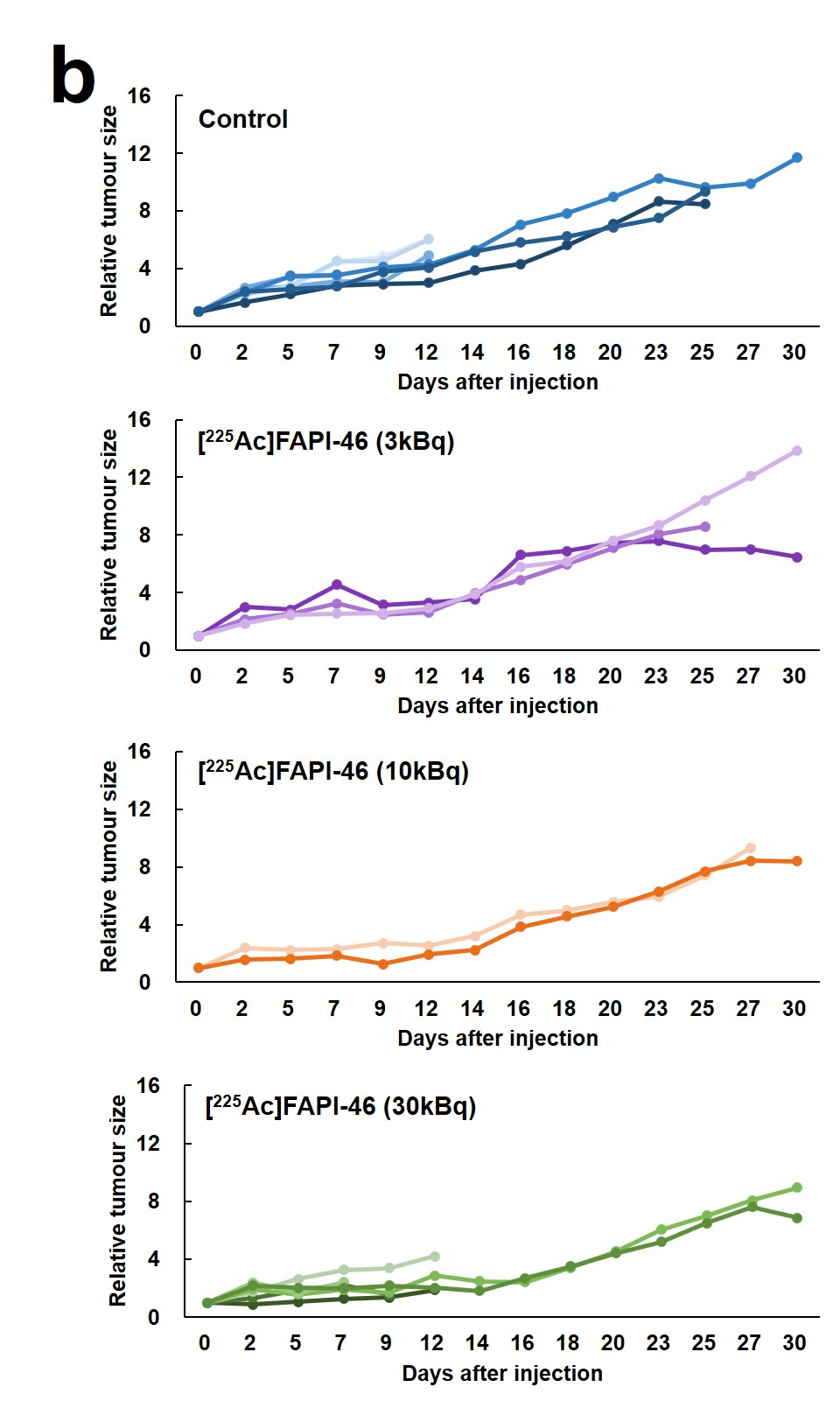

Supplement: Supplementary file 2 — Supplementary file2 (DOCX 381 KB) [file 259_2021_5554_MOESM2_ESM.docx]
